# Genetic variants in p53 signaling pathway genes predict chemotherapy efficacy in colorectal cancer

**DOI:** 10.1002/cam4.2215

**Published:** 2019-05-15

**Authors:** Ke Zhang, Yixuan Meng, Xiangming Cao, Ye Xu, Mulong Du, Yuan Wu, Lingxiang Liu

**Affiliations:** ^1^ Department of Oncology The First Affiliated Hospital of Nanjing Medical University Nanjing People's Republic of China; ^2^ The Key Laboratory of Modern Toxicology of Ministry of Education, Department of Genetic Toxicology School of Public Health, Nanjing Medical University Nanjing People’s Republic of China; ^3^ Department of Oncology The Affiliated Jiangyin Hospital of Southeast University Medical College Wuxi China; ^4^ Department of Biostatistics Nanjing Medical University Nanjing People’s Republic of China; ^5^ Department of Medical Oncology Jiangsu Cancer Hospital, Jiangsu Institute of Cancer Research, The Affiliated Cancer Hospital of Nanjing Medical University Nanjing China

**Keywords:** chemotherapy, colorectal cancer, genetic variants, survival

## Abstract

**Background:**

The murine double minute‐2 gene (*MDM2*) was originally identified as predicting chemotherapy efficacy. However, little is known regarding the association between single nucleotide polymorphisms (SNPs) in the p53 signaling pathway and prognosis/chemotherapy sensitivity in colorectal cancer.

**Methods:**

We analyzed the association between 111 SNPs in 22 p53 signaling pathway genes and both progression‐free survival (PFS) and disease control rate (DCR) using Cox regression and logistics regression analysis. The false discovery rate method was used for correction of multiple testing. Secondary structure was predicted by RNAfold. Expression qualitative trait locus analysis and mRNA expression differences were assessed using the GTEx and TCGA databases.

**Results:**

We found that the rs747828 C allele of *TP73* was significantly associated with reduced PFS (HR = 1.64, 95% CI = 1.27‐2.12, *P* = 2.00 × 10^−4^) in the additive model. In the stratified analysis, the rs747828 C allele was significantly associated with both reduced PFS (*P* = 1.40 × 10^−3^) and DCR (*P* = 1.82 × 10^−2^) in oxaliplatin‐based chemotherapy. The secondary structure of *TP73* was altered in response to different rs747828 genotypes. Although the rs747828 C allele was not associated with messenger RNA (mRNA) *TP73* expression, it was significantly associated with increased mRNA *TP73‐AS1* expression levels in sigmoid tissues. *TP73* mRNA was significantly overexpressed in tumor tissues compared to adjacent normal tissues (*P* = 2.36 × 10^−19^).

**Conclusion:**

Our findings indicate that functional genetic variants of *TP73* mediate the response to chemotherapy in colorectal cancer.

## 1 INTRODUCTION

Colorectal cancer is the second leading cause of tumor‐related mortality in men and the third in women in the United States.^1^ First‐line treatment, including fluorouracil/leucovorin combined with oxaliplatin or irinotecan, in advanced colorectal cancer has considerably improved survival.^2^, ^3^ Oxaliplatin and irinotecan are DNA‐damaging agents and are usually combined with 5‐fluorouracil as first‐line chemotherapy.^4^, ^5^ Moreover, IRI/LV/5‐FU and OXA/LV/5‐FU regimens exhibit similarly substantial efficacy.^6^

In spite of these improvements in treatment modalities, most patients eventually relapse due to the development of chemoresistance. Established predictive factors for chemotherapy efficacy include plasma mesothelin and single nucleotide polymorphisms (SNPs) in the ERCC1 gene.^7^, ^8^ Moreover, recent studies have shown that distinct patterns of gene expression are associated with both patient survival and response to chemotherapy.^9^ Key genes in the p53 signaling pathway, including murine double minute‐2 gene (*MDM2*), tumor protein p53 (*TP53*), tumor protein p63 (*TP63*), and tumor protein p73 (*TP73*), play important roles in cancer incidence, prognosis, and treatment response.^10^, ^11^, ^12^, ^13^ Moreover, *MDM2* overexpression, which results in p53 dysfunction, increases resistance to chemotherapy.^14^, ^15^, ^16^, ^17^ Furthermore, in our previous study, we found that the *MDM2* SNP309 polymorphism was a risk factor for colorectal cancers in Asians.^18^

However, few studies have reported on the predictive role of p53 signaling pathway SNPs in response to colorectal cancer chemotherapy.^19^ The magnitude of the association between chemotherapy sensitivity and p53 signaling pathway SNPs has not been thoroughly elucidated to date. To address this discrepancy, we performed a prospective study to investigate the role of p53 signaling pathway SNPs in colorectal cancer chemotherapy response.

## 2 MATERIALS AND METHODS

### 2.1 Study population

In the cohort, 344 patients with histologically diagnosed colorectal cancer were consecutively recruited from the First Affiliated Hospital of Nanjing Medical University and the Affiliated Nanjing First Hospital from September 2010 and were followed up by telephone interviews.^20^ After removing 19 patients without receiving oxaliplatin‐based or irinotecan‐based chemotherapy, a total of 325 patients were retained for further analysis (Table S1). Among them, 188 colorectal cancer patients received oxaliplatin‐based chemotherapy and 137 patients received irinotecan‐based chemotherapy. Specifically, the clinical characteristics of 166 patients with oxaliplatin‐based chemotherapy have been reported in the previous study.^21^

Peripheral whole blood samples were collected in ethylenediaminetetraacetic acid (EDTA) tubes. Clinical data including age, sex, smoking status, drinking status, tumor site, Dukes stage, tumor grade, metastatic status, chemotherapy regimen, and response to chemotherapy were also collected. Progression‐free survival (PFS) is defined as the time elapsed between chemotherapy initiation and objective disease progression, death, or last follow‐up. Written informed consent was obtained from each patient, and this study was approved by the Institutional Ethics Review Board of the Nanjing Medical University.

### 2.2 Chemotherapy regimen

All patients included in this study received oxaliplatin‐based or irinotecan‐based chemotherapy. Oxaliplatin‐based regimen consisted of a combination of oxaliplatin and short‐term infusional FU (FOLFOX) or capecitabine (XELOX). The irinotecan‐based regimen was a combination of irinotecan and short‐term infusional FU (FOLFIRI) or capecitabine (XELIRI). Therapy was continued until disease progression, unacceptable toxicity, or patient refusal.

### 2.3 Clinical evaluation

Bidimensionally measurable lesions were evaluated on CT scans before treatment and after a minimum of two cycles of chemotherapy. The primary endpoint was the tumor response to chemotherapy, evaluated according to the Response Evaluation Criteria in Solid Tumors (RECIST 1.1) as follows: (a) complete response (CR): disappearance of all target lesions; (b) partial response (PR): at least 30% decrease in the sum of the diameters of target lesions; (c) progressive disease (PD): at least 20% increase in the sum of the diameters of the target lesions; and (d) stable disease (SD): does not qualify for either PR or PD. All results were confirmed at 4 weeks. A disease control rate (DCR) was defined as the proportion of CR, PR, and SD.

### 2.4 Selection of p53 pathway‐associated genes and SNPs

Key p53 pathway‐associated genes were selected from the Kyoto Encyclopedia of Genes and Genomes (KEGG) and BioCarta (https://cgap.nci.nih.gov/Pathways/BioCarta_Pathways). Moreover, the keyword “p53 signaling pathway” was searched in PubMed (https://www.ncbi.nlm.nih.gov/pubmed) to identify p53 pathway‐associated genes. Genes located on sex chromosomes were excluded. Quality control for extracting SNPs met all the following criteria: (a) minor allelic frequency ≥0.1; (b) call rate ≥99%. Then, a pairwise linkage disequilibrium (LD) *r* ^2 ^threshold of 0.8 was used to obtain tagging SNPs with HaploView 4.2 software. SNP functions were predicted using web‐based tools, including RegulomeDB (http://regulome.stanford.edu/index), SNPinfo Web Server (http://snpinfo.niehs.nih.gov/), and HaploReg (http://archive.broadinstitute.org/mammals/haploreg/haploreg.php). We also predicted secondary structural changes caused by different tagSNP genotypes using RNAfold (http://rna.tbi.univie.ac.at/).

### 2.5 SNP genotyping

Genomic DNA was extracted from EDTA‐treated blood of all subjects using the Qiagen Blood Kit (Qiagen). Genotyping was performed using Illumina Human Qmni ZhongHua Bead Chips in all samples that met the sequencing requirements. A uniform quality control protocol was used to filter samples and SNPs.

### 2.6 Expression analysis

Expression qualitative trait locus (eQTL) analysis was performed to assess for correlations between genotypes of selected SNPs and expression levels of nearby genes using the Genotype‐Tissue Expression (GTEx) project dataset, including 203 sigmoid tissues and 246 transverse tissues. Differential gene expression of RNA‐sequencing data in colorectal cancer was analyzed from The Cancer Genome Atlas (TCGA) database (http://cancergenome.nih.gov/). Colorectal cancer tissues and normal adjacent tissues in TCGA database were used to calculate logarithmic fold change in expression levels of selected genes. All individuals included in the TCGA database were of European descent.

### 2.7 Statistical analysis

We extracted SNPs using the Han Chinese from Beijing (CHB) and Japanese from Tokyo (JPT) data from the 1000 Genomes Project and HaploView 4.2 software. Unconditional univariate and multivariate Cox regression analyses were used to calculate hazard ratios (HRs) and their 95% confidence intervals (CIs) for evaluating the association between PFS and genetic variants in colorectal cancer. To calculate the crude and adjusted odds ratios (ORs) and their 95% CIs for evaluating the correlation between DCR and genetic variants, we used an unconditional univariate and multivariate logistic regression model. The false discovery rate (FDR) method was applied for significance testing to restrict the probability of false‐positive findings in light of the large number of SNPs tested. The sequence kernel association test (SKAT) was performed to conduct gene‐based analysis. We used a two‐sided Student's *t* test to compare significant differences in gene expression between colorectal cancer tumor tissues and adjacent normal tissues. The relationship between BMI and the corresponding gene expression was examined using a linear regression model. PLINK 1.07 was used for primary statistical analysis, and other statistical analyses were performed using SAS (version 11.0; SAS Institute, Inc Cary, NC) and R software (version 3.2.3). Kaplan‐Meier curves were used to estimate the effects of identified genotypes on the cumulative probability of PFS and OS. Linkage disequilibrium (LD) between SNPs in the genes of interest was explored using LD mapping in HaploView 4.2. *P*‐values < 0.05 were considered to be statistically significant.

## 3 RESULTS

### 3.1 Characteristics of the study population

As shown in Table S1, 205 patients were male and 120 were female, with a mean age of 58.62 years being observed. Smokers and nonsmokers comprised 213 (65.54%) and 112 (34.46%) of patients, respectively. In total, 226 (69.54%) patients consumed alcohol and 99 (30.46%) patients had never consumed alcohol. Moreover, 194 (59.69%) patients suffered from colon cancer and 131 (40.31%) suffered from rectal cancer.

### 3.2 Selection of genes and SNPs from the p53 signaling pathway

**Figure 1:**
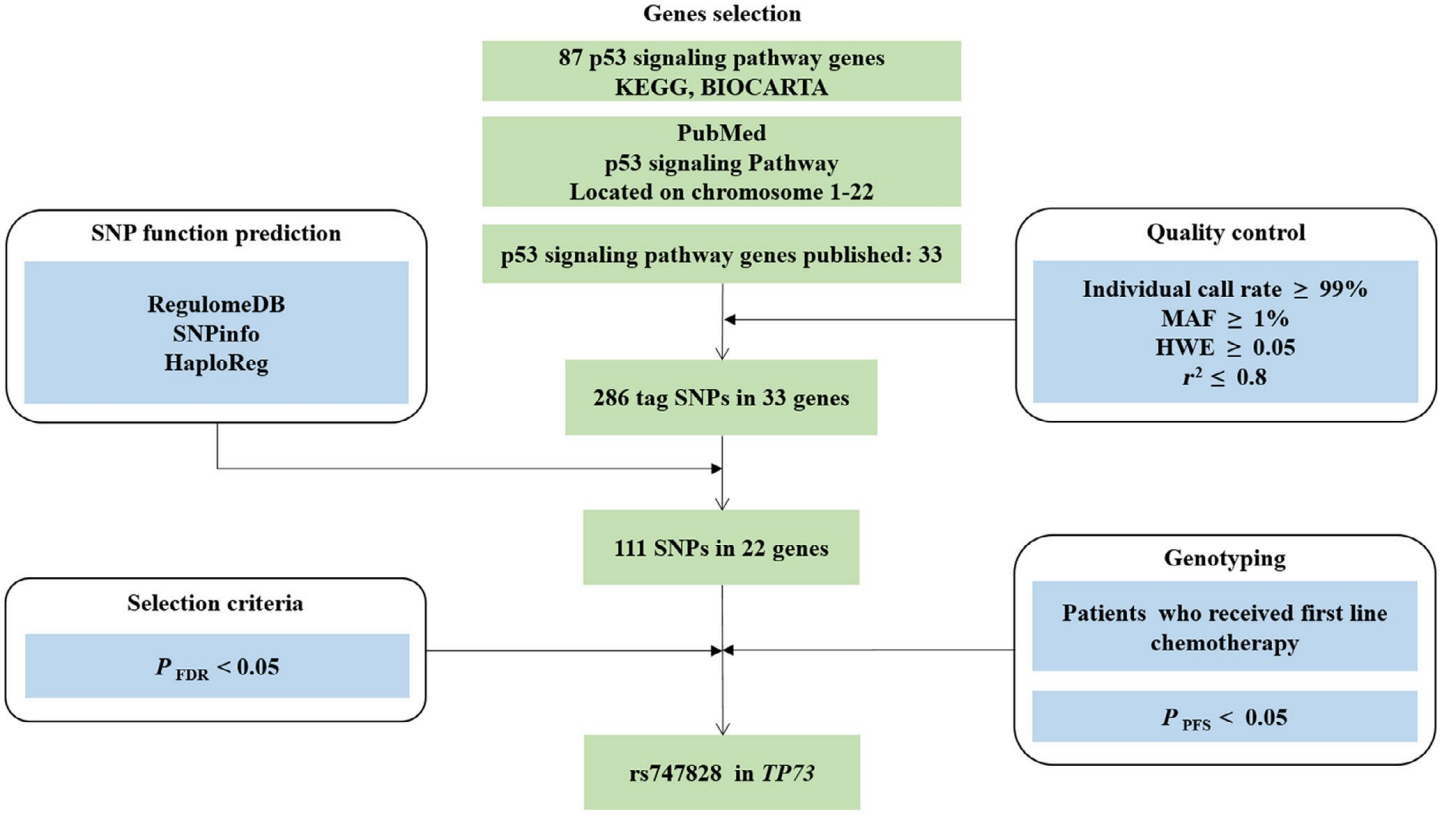
Schematic flow for selecting SNPs in the p53 signaling pathway genes. Abbreviations: HWE, Hardy‐Weinberg Equilibrium; MAF, minor allele frequency; *P*_FDR_, *P* after false discovery rate correction

As shown in Figure 1, 87 key p53 signaling pathway genes were selected from KEGG and BioCarta. To specifically investigate the association between SNPs in p53 signaling pathway genes and prognosis of patients with colorectal cancer, we identified 33 genes located on autosomal chromosomes reported by previous studies in PubMed (Table S2). Two hundred and eighty‐six SNPs were located in these 33 candidate gene regions, including 2 kb upstream. After functional annotation, 111 putative functional SNPs in 22 genes were retained in the study (Figure S1).

### 3.3 Association of SNPs with colorectal cancer prognosis

We conducted association analysis of selected SNPs with PFS of colorectal cancer. As shown in Table 1, seven SNPs (rs747828, rs2146658, rs9659688, rs3765695, rs72714570, rs3176320, and rs3176326) were significantly associated with colorectal cancer PFS in the additive model (*P* < 0.05). After FDR correction, only rs747828 in *TP73* was nominally associated with reduced PFS of colorectal cancer (adjusted HR = 1.64, 95% CI = 1.27‐2.12, *P*_FDR_ = 1.79 × 10^−2^). We further analyzed the association between selected SNPs and DCR of colorectal cancer (Table S3). Consistent with previous findings, rs747828 in *TP73* was associated with decreased DCR of colorectal cancer (adjusted OR = 1.73, 95% CI = 1.04‐2.87, *P* = 3.35 × 10^−2^). Consequently, we focused on rs747828 in *TP73* for subsequent analysis. We used four genetic models (additive, dominant, codominant, and recessive) to analyze the associations between rs747828 in *TP73* and colorectal cancer PFS and DCR (Table 2; Table S4). The SNP rs747828 was significantly associated with reduced PFS of colorectal cancer in the codominant, additive, and dominant models (adjusted HR = 1.65, 95% CI = 1.23‐2.20, *P* = 8.00 × 10^−4^; adjusted HR = 2.60, 95% CI = 1.05‐6.41, *P* = 3.82 × 10^−2^; adjusted HR = 1.64, 95% CI = 1.27‐2.12, *P* = 2.00 × 10^−4^; adjusted HR = 1.69, 95% CI = 1.27‐2.24, *P* = 3.00 × 10^−4^, respectively). In addition, rs747828 in *TP73* was associated with decreased DCR of colorectal cancer in the additive model (adjusted OR = 1.73, 95% CI = 1.04‐2.87, *P* = 3.35 × 10^−2^). However, no significant differences were observed in the analysis of association between rs747828 and PFS or DCR in the recessive model (*P* = 8.01 × 10^−2^ and *P* = 0.188, respectively).

**Table 1:** Association of seven significant SNPs and colorectal cancer PFS

SNP		Gene		MAF		Call rate		Allele^a^		PFS													
										HR		95% CI			*P*		HR^b^		95%CI		*P* b		*P*_FDR_
rs747828		*TP73*		0.159		99.08%		T/C		1.66		1.28‐2.14			**1.00 × 10** ^−^ **^4^**		1.64		1.27‐2.12		**2.00 × 10^−4^**		**1.79 × 10^−2^**
rs2146658		*TP73*		0.495		100.00%		T/G		1.28		1.06‐1.54			**9.10 × 10^−3^**		1.28		1.06‐1.54		**8.90 × 10^−3^**		0.500
rs9659688		*TP73*		0.192		99.38%		A/G		1.33		1.06‐1.67			**1.34 × 10^−2^**		1.32		1.05‐1.66		**1.63 × 10^−2^**		0.608
rs3765695		*TP73*		0.180		100.00%		C/A		1.32		1.04‐1.67			**2.48 × 10^−2^**		1.30		1.03‐1.66		**3.10 × 10^−2^**		0.870
rs72714570		*HIF1A*		0.177		100.00%		C/G		1.33		1.03‐1.72			**2.85 × 10^−2^**		1.33		1.03‐1.71		**3.12 × 10^−2^**		0.697
rs3176320		*CDKN1A*		0.217		100.00%		A/G		1.26		1.03‐1.53			**2.52 × 10^−2^**		1.25		1.02‐1.53		**3.36 × 10^−2^**		0.622
rs3176326		*CDKN1A*		0.103		100.00%		G/A		1.32		1.01‐1.73			**4.16 × 10^−2^**		1.32		1.01‐1.74		**4.60 × 10^−2^**		0.732

Note

*P* < 0.05, the values of which were presented in bold, was defined as statistically significant.

Abbreviations: CI, confidence interval; HR, hazard ratio; MAF, minor allele frequency; *P*_FDR_, *P* after false discovery rate correction; PFS, progression‐free survival.

a Reference allele/effect allele.

b Adjusted for age, sex, smoking status and drinking status in Cox regression model.

**Table 2:** Association analysis between rs747828 in *TP73* and colorectal cancer survival

Models	PFS					
	HR	95% CI	*P*	HR^a^	95% CI	*P* a
TT	1.00			1.00		
TC	1.66	1.24‐2.21	**6.00 × 10^−4^**	1.65	1.23‐2.20	**8.00 × 10^−4^**
CC	2.74	1.11‐6.74	**2.82 × 10^−2^**	2.60	1.05‐6.41	**3.82 × 10^−2^**
Additive model	1.66	1.28‐2.14	**1.00 × 10^−4^**	1.64	1.27‐2.12	**2.00 × 10^−4^**
Dominant model	1.70	1.28‐2.25	**2.00 × 10^−4^**	1.69	1.27‐2.24	**3.00 × 10^−4^**
Recessive model	2.36	0.96‐5.76	6.06 × 10^‐2^	2.23	0.91‐5.46	8.01 × 10^‐2^

*P* < 0.05, the values of which were presented in bold, was defined as statistically significant.

Abbreviations: CI, confidence interval; HR, hazard ratio; PFS, progression‐free survival.

a Adjusted for age, sex, smoking and drinking status in Cox regression model.

### 3.4 In silico analysis and gene‐based analysis

To investigate the function of selected SNPs, we performed in silico analysis using RegulomeDB, SNPinfo Web Server, and HaploReg. SNPs correlated with colorectal cancer prognosis were predicted to have active biological functions due to the integration of three online functional annotation tools (Table S5). We found that rs747828 in *TP73* possessed enhancer histone marks, altered motifs and DNAse, and its RegPotential and RegulomeDB scores were 0.102 and 5, respectively.

Furthermore, we conducted gene‐based analysis using SKAT to confirm the most significant associations between genes and colorectal cancer prognosis (Table S6). However, no significant differences were observed in association between *TP73* and prognosis in colorectal cancer patients (*P* = 0.318).

### 3.5 Stratification analysis of rs747828 in *TP73* with colorectal cancer prognosis

We further analyzed the association between rs747828 in *TP73* and colorectal cancer prognosis stratified by age, sex, smoking status, drinking status, tumor site, tumor differentiation, Dukes stage, number of metastases, and treatment in the dominant model (Table 3). We observed that the rs747828 C allele was significantly associated with reduced PFS of colorectal cancer patients with respect to drinking (adjusted HR = 2.01, 95% CI = 1.42‐2.84, *P* < 1.00 × 10^−4^), moderate and well‐differentiated tumor differentiation (adjusted HR = 1.73, 95% CI = 1.26‐2.39, *P* = 8.06 × 10^−4^), and Dukes stage D (adjusted HR = 1.73, 95% CI = 1.29‐2.31, *P* = 2.00 × 10^−4^). Interestingly, there was also a significant association between rs747828 C allele and PFS in patients who had undergone oxaliplatin‐based chemotherapy (adjusted HR = 1.85, 95% CI = 1.27‐2.70, *P* = 1.40 × 10^−3^). Furthermore, we found that the rs747828 C allele was significantly associated with decreased DCR of colorectal cancer patients with respect to age over 60 (adjusted OR = 2.61, 95% CI = 1.09‐6.26, *P* = 3.20 × 10^−2^), drinking (adjusted OR = 2.03, 95% CI = 1.01‐4.08, *P* = 4.85 × 10^−2^), rectal cancer (adjusted OR = 3.66, 95% CI = 1.35‐9.89, *P* = 1.05 × 10^−2^), Dukes stage D (adjusted OR = 1.95, 95% CI = 1.08‐3.53, *P* = 2.80 × 10^−2^), organ number of metastases over two (adjusted OR = 8.00, 95% CI = 1.43‐44.70, *P* = 1.79 × 10^−2^), and treated with oxaliplatin‐based chemotherapy (adjusted OR = 2.73, 95% CI = 1.19‐6.30, *P* = 1.82 × 10^−2^). No significant heterogeneity was observed (*P* > 0.05).

**Table 3:** *TP73* rs747828 associated with PFS and DCR in stratified analysis

Variables	Progress/Total^a^	PFS			PD/Patients^a^	DCR	
		HR^b^ (95%CI)	*P* b			OR^c^ (95%CI)	*P* c
Age							
≤60	120/165	1.65 (1.10‐2.48)	**1.54 × 10** ^−^ **^2^**		33/163	1.44 (0.64‐3.26)	0.383
>60	108/142	1.70 (1.12‐2.58)	**1.24 × 10** ^−^ **^2^**		33/139	2.61 (1.09‐6.26)	**3.20 × 10** ^−^ **^2^**
Sex							
Male	153/193	1.47 (1.02‐2.10)	**3.68 × 10** ^−^ **^2^**		43/189	1.93 (0.93‐4.00)	7.77 × 10^−2^
Female	75/114	2.14 (1.31‐3.50)	**2.40 × 10** ^−^ **^3^**		23/113	1.44 (0.53‐3.93)	0.477
Smoking status							
Positive	147/204	1.63 (1.14‐2.34)	**7.30 × 10** ^−^ **^3^**		39/201	1.65 (0.78‐3.49)	0.190
Negative	81/103	1.81 (1.11‐2.96)	**1.69 × 10** ^−^ **^2^**		27/101	2.07 (0.79‐5.46)	0.141
Drinking status							
Positive	155/217	2.01 (1.42‐2.84)	**<1.00 × 10** ^−^ **^4^**		44/214	2.03 (1.01‐4.08)	**4.85 × 10** ^−^ **^2^**
Negative	73/90	1.17 (0.71‐1.93)	0.546		22/88	1.34 (0.46‐3.87)	0.594
Tumor site							
Colon	133/185	1.80 (1.22‐2.64)	**2.85 × 10** ^−^ **^3^**		41/182	1.23 (0.56‐2.67)	0.607
Rectum	95/122	1.65 (1.06‐2.57)	**2.62 × 10** ^−^ **^2^**		25/120	3.66 (1.35‐9.89)	**1.05 × 10** ^−^ **^2^**
Tumor differentiation							
Moderate and well	178/240	1.73 (1.26‐2.39)	**8.06 × 10** ^−^ **^4^**		50/235	1.83 (0.93‐3.59)	7.86 × 10^−2^
Poor	50/67	1.45 (0.76‐2.77)	0.265		16/67	1.81 (0.52‐6.35)	0.353
Dukes stage							
C	13/23	3.30 (0.37‐ 29.35)	0.284		4/21	0.15 (NA)	0.993
D	215/284	1.73 (1.29‐2.31)	**2.00 × 10** ^−^ **^4^**		62/281	1.95 (1.08‐3.53)	**2.80 × 10** ^−^ **^2^**
Metastasis							
≤2	175/224	1.50 (1.08‐2.07)	**1.52 × 10^‐2^**		50/224	1.29 (0.65‐2.55)	0.463
>2	36/47	3.40 (1.40‐8.26)	**7.00 × 10** ^−^ **^3^**		12/47	8.00 (1.43‐44.70)	**1.79 × 10** ^−^ **^2^**
Treatment							
Oxaliplatin	124/177	1.85 (1.27‐2.70)	**1.40 × 10** ^−^ **^3^**		30/173	2.73 (1.19‐6.30)	**1.82 × 10** ^−^ **^2^**
Irinotecan	104/130	1.49 (0.96‐2.32)	7.69 × 10^−2^		36/129	1.25 (0.53‐2.96)	0.612

*P* < 0.05, the values of which were presented in bold, was defined as statistically significant.

Abbreviations: CI, confidence interval; DCR, disease control rate; HR, hazard ratio; OR, odds ratio; PD, progressive disease; PFS, progression‐free survival.

a Some cases were not included due to missing clinical data or genotyping.

b Adjusted for age, sex, smoking and drinking status in Cox regression model.

c Adjusted for age, sex, smoking and drinking status in logistic regression model.

It is worth noting that the chemotherapy regimen was an effect modifier between *TP73* rs747828 and colorectal cancer prognosis. We analyzed the relationship of *TP73* rs747828 with PFS and DCR stratified by treatments (Table S7). In the oxaliplatin‐based chemotherapy subgroup, results of the additive and dominant models indicated that the rs747828 C allele is associated with reduced PFS and decreased DCR. The effect of the TC genotype was significantly associated with reduced PFS and decreased DCR in the codominant model (adjusted HR = 1.86, 95% CI = 1.27‐2.71, *P* = 1.30 × 10^−3^; adjusted HR = 2.80, 95% CI = 1.21‐6.44, *P* = 1.59 × 10^−2^, respectively). Due to the limited sample size, results could not be calculated in the codominant (CC genotype) or recessive models.

**Figure 2:**
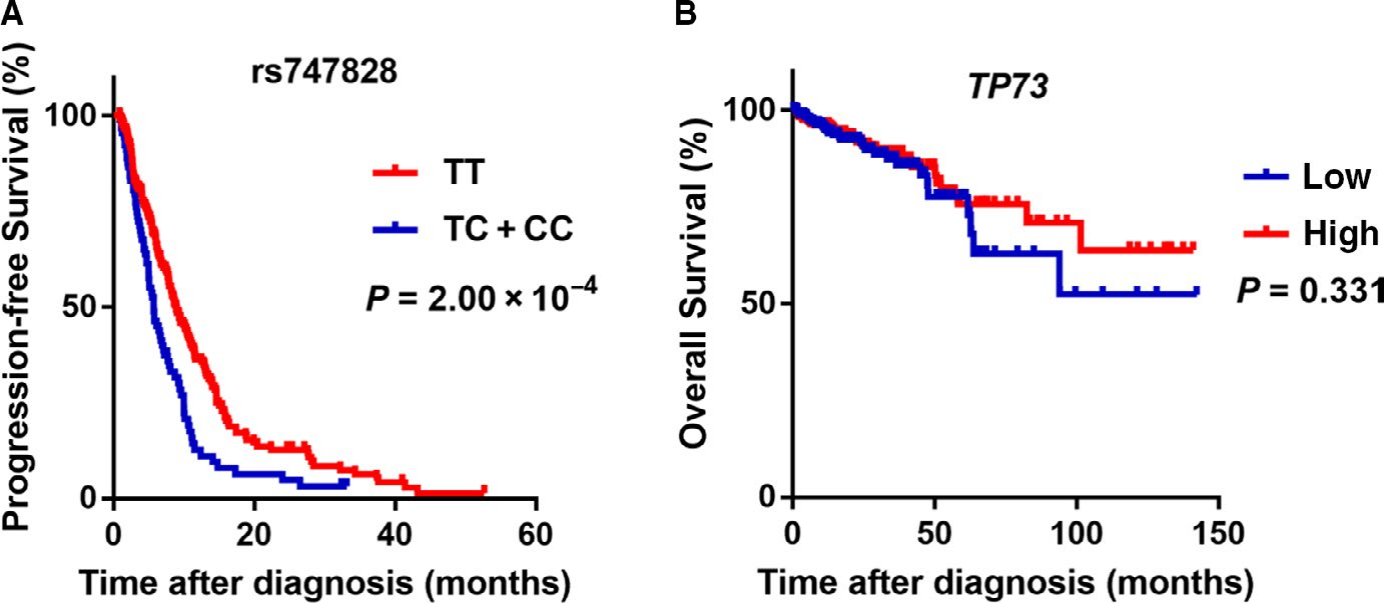
Kaplan‐Meier curves in patients with colorectal cancer. A, Kaplan‐Meier curves of progression‐free survival (PFS) for rs747828 using Cox regression in colorectal cancer patients. The starting point of PFS was the first date of chemotherapy instead of the date of diagnosis. B, Kaplan‐Meier curves of OS for *TP73* expression level using Cox regression in colorectal cancer patients

In the irinotecan‐based chemotherapy subgroup, results of the additive model indicated that the rs747828 C allele was associated with reduced PFS and decreased DCR (adjusted HR = 1.46, 95% CI = 1.02‐2.10, *P* = 1.03 × 10^−2^; adjusted HR = 2.49, 95% CI = 1.12‐5.55, *P* = 2.55 × 10^−2^, respectively). However, the rs747828 C allele was neither associated with reduced PFS nor decreased DCR in the dominant model after adjusting for age, sex, smoking, and drinking status. We also examined Kaplan‐Meier curves of PFS for rs747828 in patients suffering from colorectal cancer in the dominant model (Figure 2A). Patients with the TC/CC rs747828 genotypes exhibited reduced PFS compared to patients with the TT genotype.

### 3.6 Prediction of rs747828 *TP73* folding structures and eQTL analysis

We conducted in silico analysis using RNAfold to predict the *TP73* secondary structure of rs747828. Results showed that the secondary structure was dramatically altered in rs747828 T/C alleles (Figure S2), with the minimum free energy decreasing from −13.90 kcal/mol to −16.00 kcal/mol. We further conducted eQTL analysis to evaluate the effect of rs747828 in *TP73*. No significant association was observed between rs747828 and *TP73* in the GTEx or TCGA databases (Figure S3A‐B). Moreover, no significant difference was found in 246 colon‐transverse samples (NES = 0.020, *P* = 0.786), but rs7474828 was significantly correlated with the expression of *TP73‐AS1* in 203 colon‐sigmoid samples (NES = 0.369, *P* = 1.35 × 10^−3^) (Figure S3C).

### 3.7 Gene expression analysis in colorectal tumors and adjacent normal tissues

**Figure 3:**
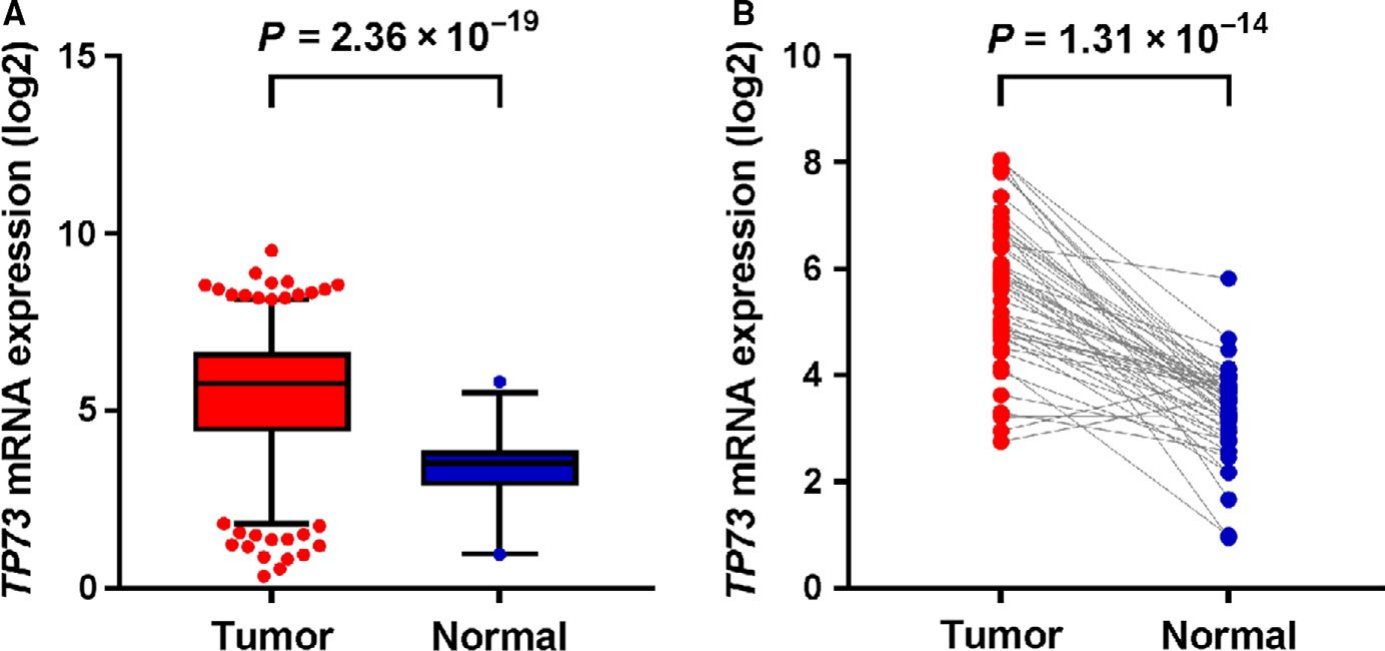
The expression of *TP73* in colorectal cancer tissues and normal adjacent tissues in TCGA database. A, no pairing. B, Pairing using student's *t* test

We used TCGA database to analyze differential expression of *TP73* between adjacent normal and tumor tissues. In Figure 3, we observed that *TP73* expression was significantly increased in TCGA database (*P* = 2.36 × 10^−19^). Among these tissues, *TP73* expression was significantly different (*P* = 1.31 × 10^−14^) in paired tumor tissues and adjacent normal tissues. Furthermore, we analyzed differential expression of *TP73* in colorectal cancer tissues based on age, sex, site, and KRAS mutation status (Figure S4).

We also examined differential expression of *TP73* in colorectal cancer tissues based on major cancer stages, metastasis, and BMI (Figure S5). Each stage and metastasis were significantly different (all *P* < 1.00 × 10^−4^). Moreover, *TP73* expression was decreased in BMI over 27 (*P* = 3.25 × 10^−2^), and *TP73* expression decreased with increasing BMI (*P* = 4.00 × 10^−3^, *r* ^2^ = 0.027) by linear regression analysis. Finally, we observed that no significant association existed between *TP73* expression levels and overall survival in patients suffering from colorectal cancer (Figure 2B).

## 4 DISCUSSION

Predictive markers for chemotherapy resistance are highly useful to prospectively identify patients who will benefit from the treatment. Previous studies have suggested that genetic variants are associated with prognosis for patients suffering from metastatic colorectal cancer who are treated with oxaliplatin‐based chemotherapy.^22^ Several reports indicate that SNPs related to chemotherapy resistance exist in p53 signaling pathway genes.^23^, ^24^, ^25^ Most studies have shown that SNPs affect cancer prognosis by altering messenger RNA (mRNA) expression or by combining with microRNA.^26^ However, the mechanisms whereby SNPs reportedly affect mRNA are inconsistent. Several studies failed to find mutations in the *TP73* gene, suggesting that its mutation plays little role in tumor progression.^27^ In this study, we evaluated the relationship between SNPs in p53 signaling pathway genes and colorectal cancer prognosis in a Chinese population. We observed that the *TP73* rs747828 C allele might predict reduced PFS and decreased DCR. In addition, rs747828 altered *TP73‐AS1* expression and affects the secondary structure of *TP73*. Collectively, our study provides evidence of the relationship between *TP73* genetic variants and prognosis in colorectal cancer.

Although rs747828 does not affect mRNA *TP73* expression, it was significantly associated with mRNA *TP73‐AS1* expression levels in sigmoid tissues. In particular, *TP73‐AS1* is the antisense of the coding gene *TP73*, which encodes a protein sharing notable similarities to *TP53* in structure and function.^28^ Previous studies reported that global genomic analysis indicates the transformation of the antisense RNA can affect expression of the sense gene, and SNPs may functionally regulate mRNA expression.^29^ Given that *TP73‐AS1* mantles substantial portions of *TP73*, *TP73‐AS1* may function through posttranscriptional regulation of *TP73*.^30^ Therefore, rs747828 may affect *TP73* by altering *TP73‐AS1* expression. Of note, the mutation frequency of rs747828 was only 0.01 in both American and European samples, while it was 0.16 in Asian samples. However, research employing the TCGA database helps to elucidate the state of American and European populations. Furthermore, functional biological experiments are necessary to validate our findings in the future.

Several studies have reported that alcohol consumption is a negative prognostic factor in colorectal cancer, but the results have been inconsistent.^31^, ^32^, ^33^ In the present study, rs747828 was robustly associated with PFS and DCR in the drinking subgroup. These differences might be caused by population heterogeneity to some extent. Furthermore, a previous study reported that irinotecan and oxaliplatin regimens have similar chemotherapy efficacy.^6^ However, oxaliplatin‐based chemotherapy resulted in superior DCR and overall survival compared with irinotecan‐based chemotherapy in a meta‐analysis.^34^ Obviously, these studies did not take different genotypes into account in their analysis. In our study, rs747828 was significantly associated with reduced PFS and decreased DCR in oxaliplatin regimens but not in irinotecan regimens by stratified analysis. Hence, we provided evidence that genotype influences treatment effect, and different chemotherapy drugs possess diverse therapeutic targets and mechanisms.

In conclusion, our study provides a new view of the development of biomarkers for predicting chemotherapeutic efficacy in colorectal cancer. Genetic variants in *TP73* may predict chemotherapy sensitivity of colorectal cancer patients treated with oxaliplatin‐based chemotherapy. Moreover, increased *TP73* mRNA expression was observed in colorectal tumor tissues compared to corresponding normal tissues.

## CONFLICT OF INTEREST

The authors indicated no potential conflict of interest.
