# Supplementary material for: Genetic variants in p53 signaling pathway genes predict chemotherapy efficacy in colorectal cancer
Source: Cancer Med. 2019 May 15;8(7):3428–36. doi: 10.1002/cam4.2215 (PMC6601596; doi:10.1002/cam4.2215)

**Supplementary Figure Legends**

**Fig. S1. Key genes of the p53 signaling pathway.**

**Fig. S2. In *silico* prediction of rs747828 folding structures changed corresponding to rs747828 T to C allele.**

The arrows indicated the changes in structure caused by rs747828.

**Fig. S3. The eQTL analysis in GTEx database and TCGA database.**

The eQTL analysis between rs747828 and *TP73* in GTEx database (a) and in TCGA database (b) and *TP73-AS1* in colon-Sigmoid and Colon-Transverse in GTEx database (c). NES: the slope of the linear regression of normalized expression data versus the three genotype categories using single-tissue eQTL analysis, representing eQTL effect size; the normalized expression values are based on quantile normalization within each tissue, followed by inverse quantile normalization for each gene across samples.

**Fig. S4. Expression of *TP73* in colorectal cancer tissues stratified by age, sex, site and *KRAS*.**

**Fig. S5. Expression of *TP73* in colorectal cancer tissues stratified by major cancer stages (a), metastasis (b) and BMI (c-d).**

One record was deleted because it might be reversed between height and weight.

**P* < 0.0001.

**Table S1. The characteristics of patients with colorectal cancer.**

| **Variables** | **Total, n (%) or Mean (SD^a^)** |
| --- | --- |
| Age (mean ± SD) | 58.62 ± 12.24 |
| Sex |  |
| Male | 205 (63.08) |
| Female | 120 (36.92) |
| Smoking status |  |
| Positive | 213 (65.54) |
| Negative | 112 (34.46) |
| Drinking status |  |
| Positive | 226 (69.54) |
| Negative | 99 (30.46) |
| Tumor site |  |
| Colon | 194 (59.69) |
| Rectum | 131 (40.31) |
| Dukes stage |  |
| C | 23 (7.08) |
| D | 302 (92.92) |
| Tumor grade |  |
| Well and moderate | 255 (78.46) |
| Poor | 70 (21.54) |
| Metastasis^b^ |  |
| ≤2 | 237 (82.01) |
| >2 | 52 (17.99) |
| Treatment |  |
| Oxaliplatin-based | 188 (57.85) |
| Irinotecan-based | 137 (42.15) |
| Response to chemotherapy^b^ |  |
| PD | 66 (21.64) |
| SD | 183 (60.00) |
| PR | 55 (18.03) |
| CR | 1 (0.33) |

^a^SD, standard deviation.

^b^Some patients were excluded due to missing clinical data.

PD, progressive disease; SD, stable disease.

PR, partial response; CR, complete response.

**Table S2. Key genes selected of the p53 signaling pathway.**

| **Genes** | **Location^a^** |
| --- | --- |
| *MDM4* | Chr 1: 204485507-204596131 |
| *RPL11* | Chr 1: 24018269-24022915 |
| *RPL5* | Chr 1: 93297594-93307481 |
| *ASF1A* | Chr 6: 119215241-119230336 |
| *EZR* | Chr 6: 159186773-159240456 |
| *SHH* | Chr 7: 155595558-155604967 |
| *CDKN2A* | Chr 9: 21967751-21994490 |
| *PTEN* | Chr 10: 89623195-89728532 |
| *SP1* | Chr 12: 53773979-53810230 |
| *RB1* | Chr 13: 48877883-49056026 |
| *AKT1* | Chr 14: 105235686-105262080 |
| *HIF1A* | Chr 14: 62162118-62214977 |
| *RPL4* | Chr 15: 66791653-66816870 |
| *USP10* | Chr 16: 84733555-84813528 |
| *USP7* | Chr 16: 8985951-9057341 |
| *RPL23* | Chr 17: 37006321-37010053 |
| *BCL2* | Chr 18: 60790579-60987011 |
| *PDCD5* | Chr 19: 33072071-33078358 |
| *E2F1* | Chr 20: 32263292-32274210 |
| *NFATC2* | Chr 20: 50003494-50179370 |
| *XBP1* | Chr 22: 29190548-29196560 |
| *ATR* | Chr 3: 142168077-142297668 |
| *HIPK2* | Chr 7: 139415730-139477693 |
| *HIPK2* | Chr 7: 139246316-139342363 |
| *ATM* | Chr 11: 108093559-108239829 |
| *TP73* | Chr 1: 3569129-3652765 |
| *TP53* | Chr 17: 7571720-7590868 |
| *ABL1* | Chr 9: 133588266-133763062 |
| *GADD45* | Chr 1: 68150860-68154021 |
| *JUN* | Chr 1: 59246463-59249785 |
| *APAF1* | Chr 12: 99039078-99129211 |
| *CDKN1A* | Chr 6: 36644237-36655116 |
| *MDM2* | Chr 12: 69201952-69239324 |

^a^Based on NCBI build 37 of the human genomes.

**Table S3. Association of seven significant SNPs and colorectal cancer DCR in additive model.**

|  | |  |  |  | **DCR** | | | | | |
| --- | --- | --- | --- | --- | --- | --- | --- | --- | --- | --- |
| **SNP** | **Gene** | **MAF** | **Call rate** | **Allele^a^** | **OR** | **95% CI** | ***P*** | **OR^b^** | **95%CI** | ***P*^b^** |
| rs747828 | *TP73* | 0.159 | 99.08% | T/C | 1.72 | 1.05-2.84 | **3.28×10^-2^** | 1.73 | 1.04-2.87 | **3.35×10^-2^** |
| rs2146658 | *TP73* | 0.495 | 100.00% | T/G | 1.32 | 0.90-1.93 | 0.152 | 1.31 | 0.90-1.92 | 0.159 |
| rs9659688 | *TP73* | 0.192 | 99.38% | A/G | 1.28 | 0.81-2.03 | 0.294 | 1.27 | 0.80-2.03 | 0.312 |
| rs3765695 | *TP73* | 0.18 | 100.00% | C/A | 1.02 | 0.63-1.66 | 0.934 | 1.03 | 0.63-1.69 | 0.899 |
| rs72714570 | *HIF1A* | 0.177 | 100.00% | C/G | 1.52 | 0.92-2.50 | 0.101 | 1.54 | 0.93-2.55 | 9.21×10^-2^ |
| rs3176320 | *CDKN1A* | 0.217 | 100.00% | A/G | 1.36 | 0.89-2.07 | 0.154 | 1.35 | 0.88-2.07 | 0.176 |
| rs3176326 | *CDKN1A* | 0.103 | 100.00% | G/A | 1.84 | 1.07-3.17 | **2.85×10^-2^** | 1.79 | 1.03-3.11 | **3.85×10^-2^** |

^a^Reference allele/effect allele.

^b^Adjusted for age, sex, smoking status and drinking status in Logistic regression model.

MAF, minor allele frequency.

DCR, disease control rate.

PD, progressive disease.

OR, odds ratio.

CI, confidence interval.

*P* < 0.05, the values of which were presented in bold, was defined as statistically significant.

**Table S4. Association between rs747828 and response to chemotherapy.**

|  | **DCR** | | | | | |
| --- | --- | --- | --- | --- | --- | --- |
| **Models** | **OR** | **95% CI** | ***P*** | **OR^a^** | **95% CI** | ***P*^a^** |
| TT | 1 |  |  | 1 |  |  |
| TC | 1.69 | 0.93-3.07 | 8.72×10^-2^ | 1.69 | 0.92-3.08 | 9.01×10^-2^ |
| CC | 3.22 | 0.69-14.94 | 0.136 | 3.29 | 0.70-15.59 | 0.133 |
| Additive model | 1.72 | 1.05-2.84 | **3.28×10^-2^** | 1.73 | 1.04-2.87 | **3.35×10^-2^** |
| Dominant model | 1.79 | 1.00-3.19 | **4.84×10^-2^** | 1.79 | 1.00-3.20 | 5.00×10^-2^ |
| Recessive model | 2.76 | 0.60-12.66 | 0.191 | 2.82 | 0.60-13.18 | 0.188 |

^a^Adjusted for age, sex, smoking and drinking status in Logistic regression model.

DCR, disease control rate.

PD, progressive Disease.

OR, odds ratio.

*P* < 0.05, the values of which were presented in bold, was defined as statistically significant.

**Table S5. In *silico* analysis for seven SNPs function annotation.**

| **SNP** | **Chr: Position^a^** | **Gene** | **Allele^b^** | **MAF^c^** | **Score^d^** | **RP^e^** | **HaploReg** |
| --- | --- | --- | --- | --- | --- | --- | --- |
| rs747828 | 1: 3636226 | *TP73* (intronic) | T/C | 0.159 | 5 | 0.102 | Enhancer histone marks, Motifs changed, DNAse |
| rs2146658 | 1: 3637613 | *TP73* (intronic) | T/G | 0.495 | 5 | 0.008 | Enhancer histone marks, Motifs changed |
| rs9659688 | 1: 3649805 | *TP73* (3'-UTR) | A/G | 0.192 | 4 | 0.141 | Enhancer histone marks, Motifs changed, DNAse, Promoter histone marks |
| rs3765695 | 1: 3587377 | *TP73* (intronic) | C/A | 0.180 | 5 | NA | Enhancer histone marks, Proteins bound, Motifs changed |
| rs72714570 | 14: 62196617 | *HIF1A* (intronic) | C/G | 0.177 | 5 | NA | Enhancer histone marks, , DNAse |
| rs3176320 | 6: 36646788 | *CDKN1A* (intronic) | A/G | 0.217 | 4 | 0.299 | Enhancer histone marks, Motifs changed, DNAse, Promoter histone marks, Proteins bound, GRASP QTL hits, Selected Eqtl hits |
| rs3176326 | 6: 36647289 | *CDKN1A* (intronic) | G/A | 0.103 | 4 | 0.311 | Enhancer histone marks, Motifs changed, DNAse, Promoter histone marks, Proteins bound, Selected Eqtl hits |

^a^Based on NCBI build 37 of the human genomes.

^b^Reference allele/effect allele.

^c^Minor allele frequency.

^d^Based on RegulomeDB.

^e^RP, RegPotential, Based on SNPinfo Web Server.

NA, not available.

**Table S6. Gene-based analysis results by SKAT.**

| **Gene** | ***P*** | **Q** |
| --- | --- | --- |
| *ABL1* | 0.818 | 7.290 |
| *APAF1* | 0.046 | 20.149 |
| *ASF1A* | 0.515 | 1.965 |
| *ATR* | 0.624 | 1.215 |
| *BCL2* | 0.827 | 53.207 |
| *CDKN1A* | 0.055 | 78.722 |
| *CDKN2A* | 0.771 | 0.141 |
| *EZR* | 0.645 | 0.935 |
| *HIF1A* | 0.189 | 46.399 |
| *HIPK2* | 1.000 | 0.559 |
| *JUN* | 0.319 | 5.351 |
| *MDM4* | 0.601 | 11.257 |
| *NFATC2* | 0.998 | 63.089 |
| *PDCD5* | 0.301 | 5.040 |
| *RB1* | 0.809 | 0.118 |
| *RPL11* | 0.851 | 0.016 |
| *RPL23* | 0.414 | 19.242 |
| *RPL4* | 0.642 | 4.082 |
| *TP53* | 0.384 | 3.926 |
| *TP73* | 0.318 | 57.387 |
| *USP10* | 0.463 | 24.698 |
| *USP7* | 0.429 | 3.123 |

**Table S7. SNP rs747828 associated with PFS and DCR in analysis stratified by treatment.**

|  | **PFS** | | |  | **DCR** | | |
| --- | --- | --- | --- | --- | --- | --- | --- |
| **Treatment** | **HR^a^** | **95% CI** | ***P*** |  | **OR^a^** | **95% CI** | ***P*** |
| Oxaliplatin |  |  |  |  |  |  |  |
| TT | 1 |  |  |  | 1 |  |  |
| TC | 1.86 | 1.27-2.71 | **1.30×10^-3^** |  | 2.80 | 1.21-6.44 | **1.59×10^-2^** |
| CC | NA | | |  | NA | | |
| Additive model | 1.83 | 1.26 -2.66 | **1.60×10^-3^** |  | 2.49 | 1.12-5.55 | **2.55×10^-2^** |
| Dominant model | 1.85 | 1.27-2.70 | **1.40×10^-3^** |  | 2.73 | 1.19-6.30 | **1.82×10^-2^** |
| Recessive model | NA | | |  | NA | | |
| Irinotecan |  |  |  |  |  |  |  |
| TT | 1 |  |  |  | 1 |  |  |
| TC | 1.40 | 0.87-2.23 | 0.163 |  | 1.12 | 0.43-2.90 | **1.59×10^-2^** |
| CC | 2.35 | 0.92-5.95 | 7.27×10^-2^ |  | 2.80 | 1.21-6.44 | 0.991 |
| Additive model | 1.46 | 1.02-2.10 | **1.03×10^-2^** |  | 2.49 | 1.12-5.55 | **2.55×10^-2^** |
| Dominant model | 1.49 | 0.96-2.32 | 7.69×10^-2^ |  | 1.25 | 0.53-2.96 | 0.612 |
| Recessive model | 2.12 | 0.85-5.32 | 0.108 |  | NA | | |

^a^Adjusted for covariates (age, sex, smoking, drinking).

NA, results could not be calculated due to fewer samples.

*P* < 0.05, the values of which were presented in bold, was defined as statistically significant.

**Fig. S1**

**
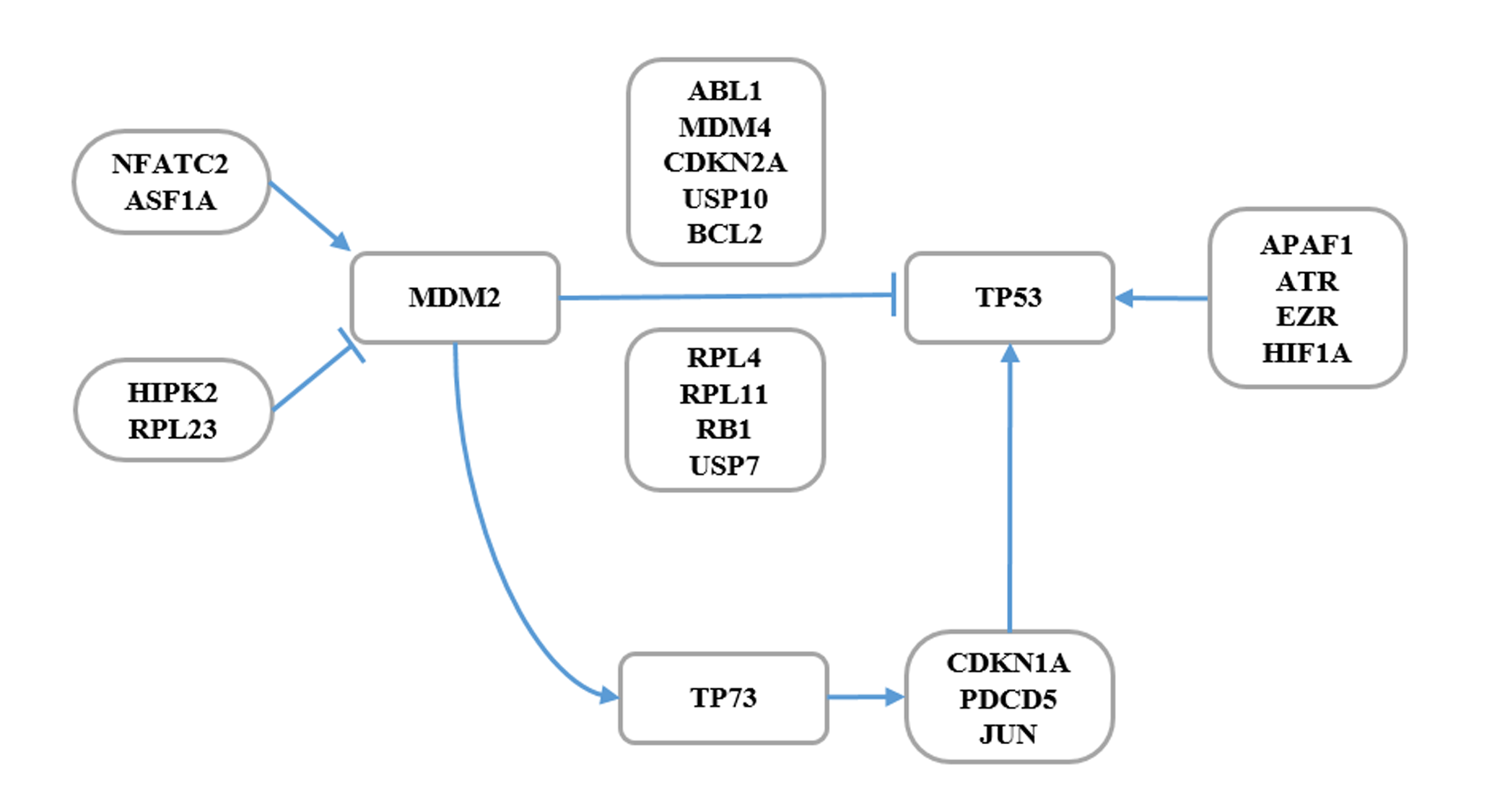
**

**Fig. S2**


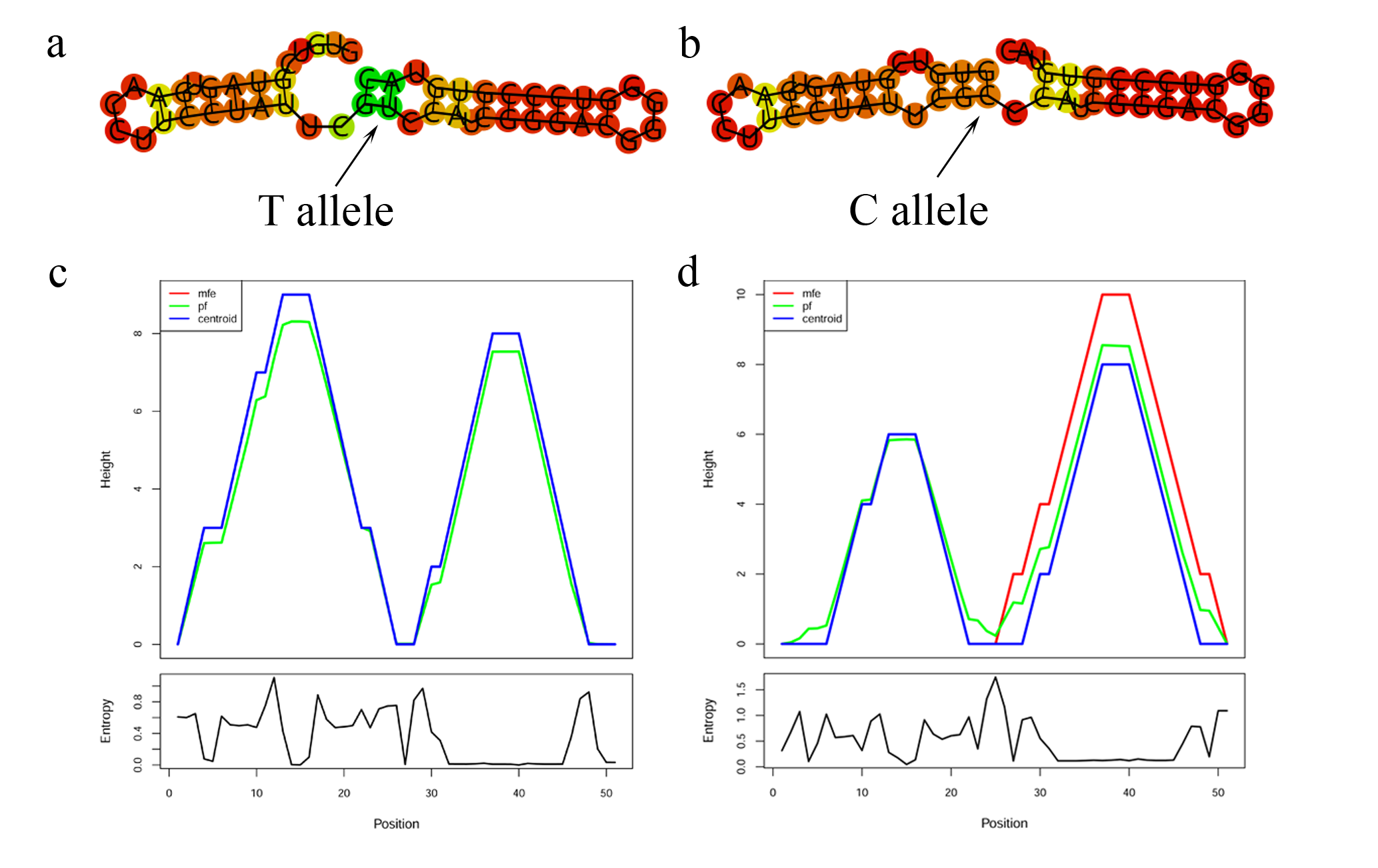


**Fig. S3**


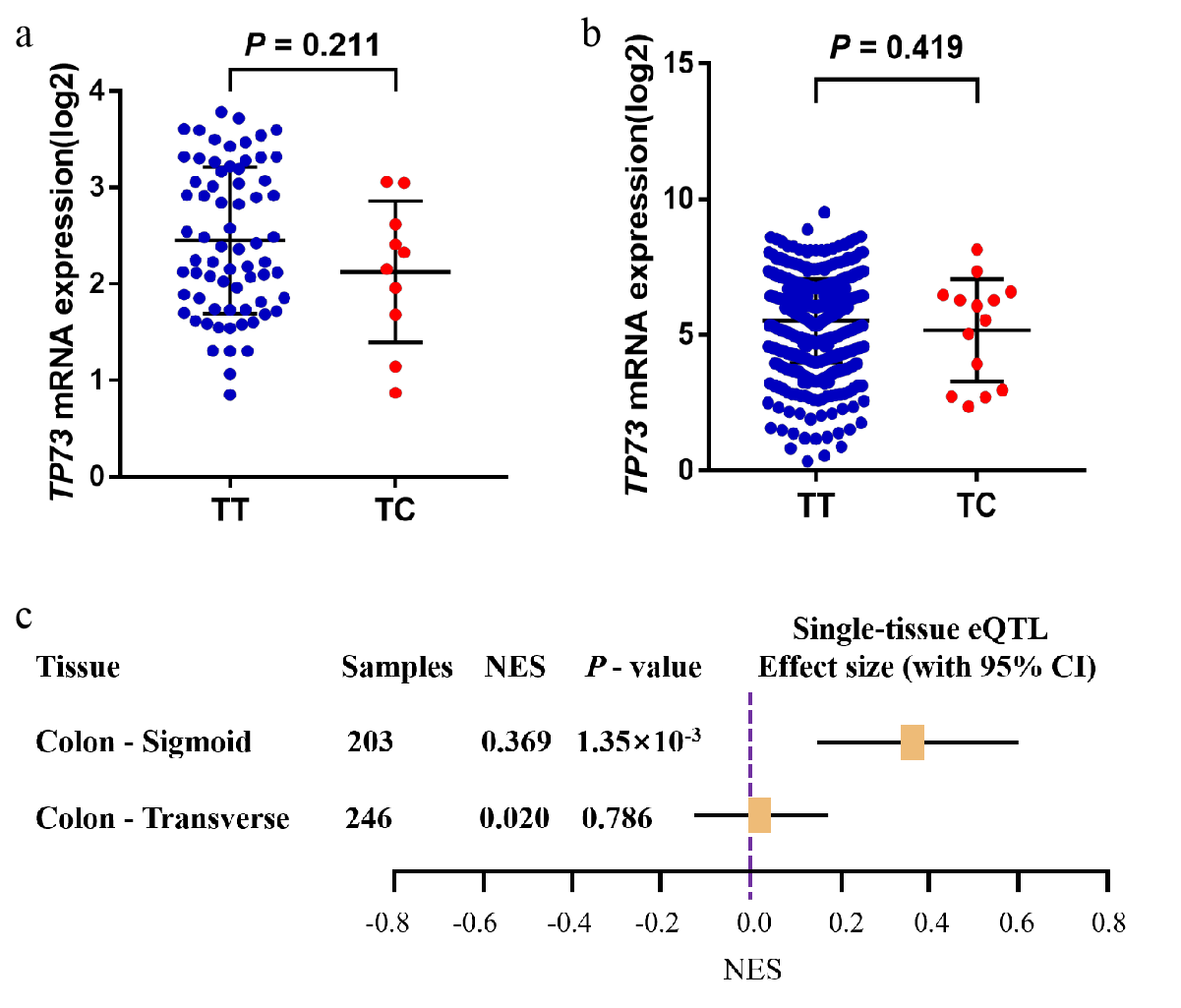


**Fig. S4**


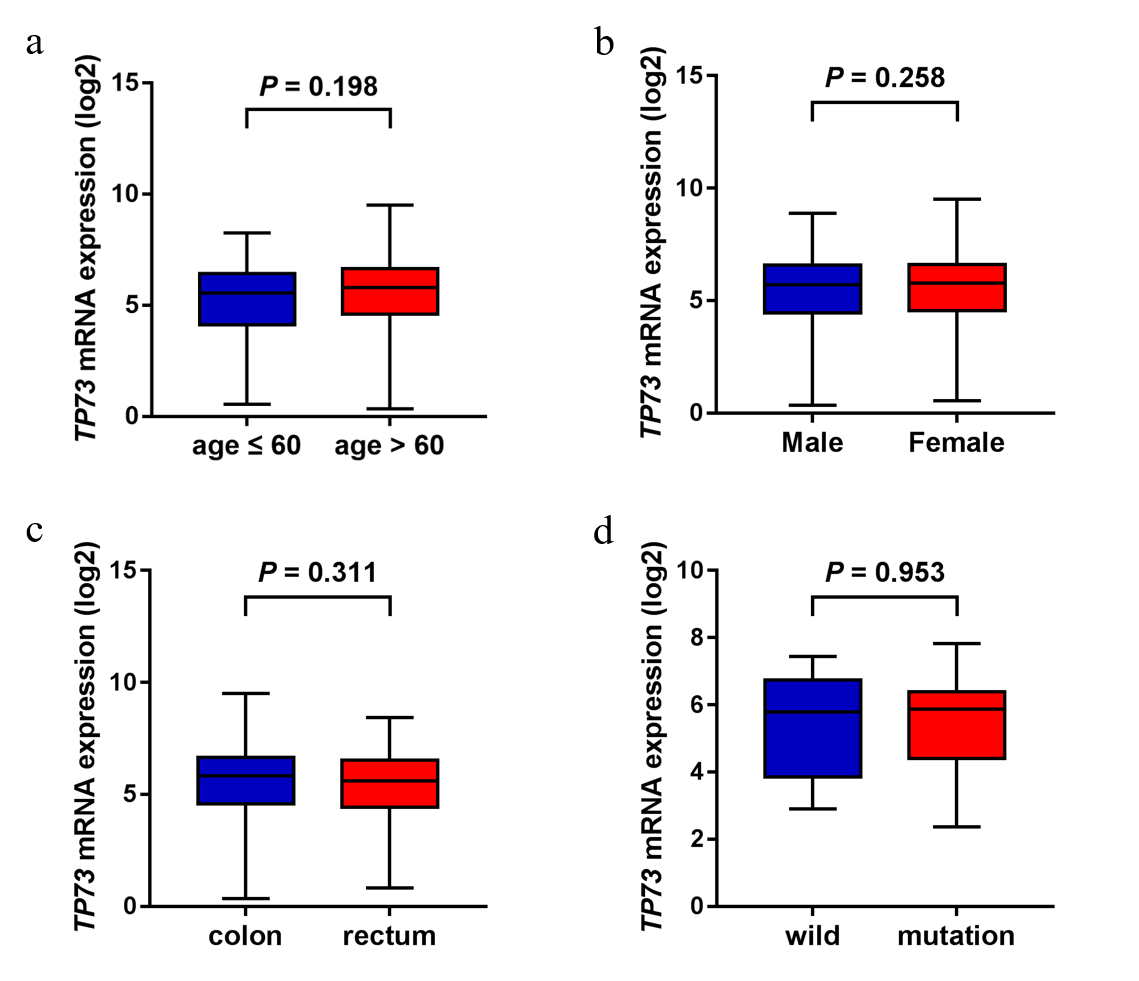


**Fig. S5**


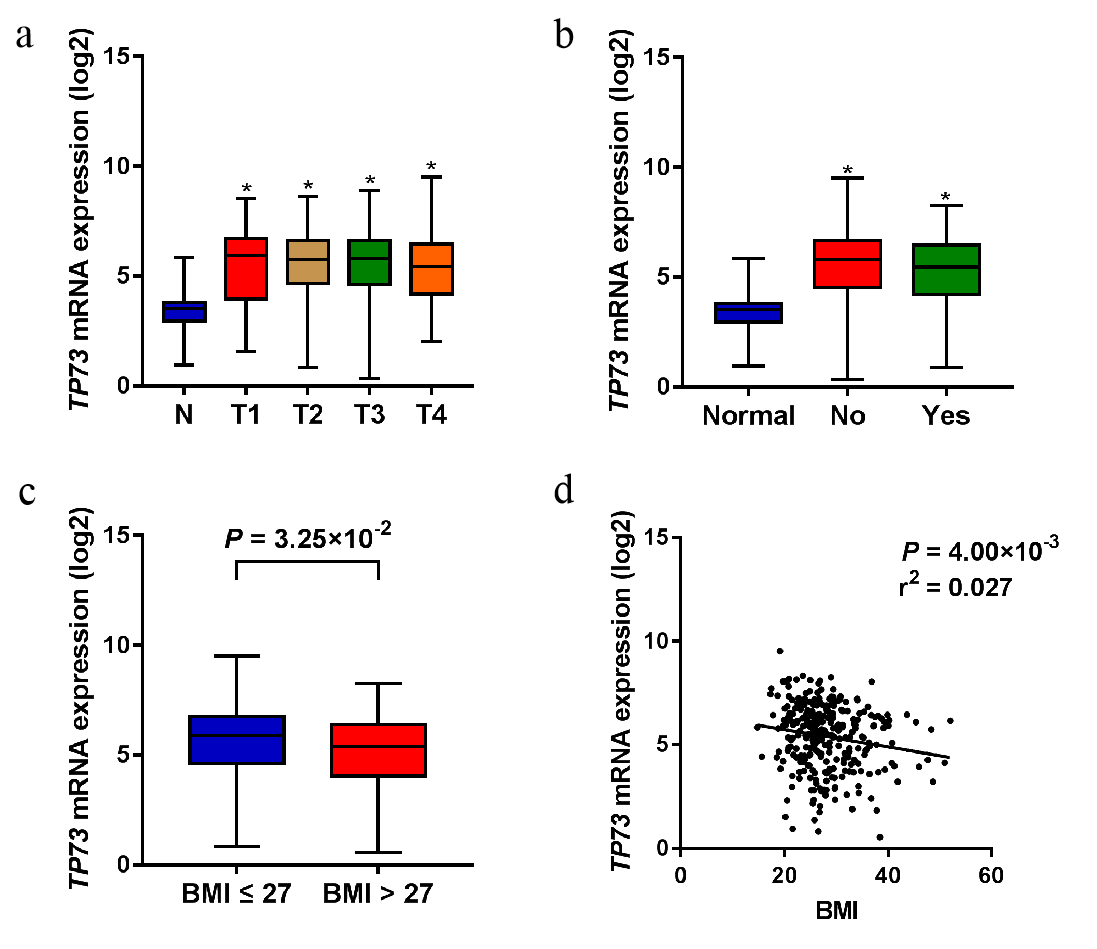

Supplement: Supplementary file 1 [file CAM4-8-3428-s001.docx]
